# Patient safety in prisons: a multi-method analysis of reported incidents in England

**DOI:** 10.1177/01410768231166138

**Published:** 2023-05-17

**Authors:** Isobel J McFadzean, Kate Davies, Thomas Purchase, Adrian Edwards, Stuart Hellard, Darren M Ashcroft, Anthony J Avery, Sandra Flynn, Tom Hewson, Melanie Jordan, Richard Keers, Maria Panagioti, Verity Wainwright, Florian Walter, Jenny Shaw, Andrew Carson-Stevens

**Affiliations:** 1 Division of Population Medicine, 2112 School of Medicine, Cardiff University, Cardiff, CF14 4YU, UK; 2 PRIME Centre Wales, Division of Population Medicine, 2112 School of Medicine, Cardiff University, Cardiff, CF14 4YU, UK; 3 Centre for Pharmacoepidemiology and Drug Safety, Division of Pharmacy and Optometry, School of Health Sciences, University of Manchester, Manchester, M13 9PL, UK; 4 NIHR Greater Manchester Patient Safety Translational Research Centre (PSTRC), University of Manchester, Manchester, M13 9PL, UK; 5 Centre for Academic Primary Care, School of Medicine, University of Nottingham, Nottingham, NG7 2UH, UK; 6 Faculty of Biology, Medicine and Health, Centre for Mental Health and Safety, University of Manchester, Manchester, M13 9PL, UK; 7 School of Sociology & Social Policy, University of Nottingham, Nottingham, NG7 2RD, UK; 8 Greater Manchester Mental Health NHS Foundation Trust (GMMH), Manchester, M25 3BL, UK; 9 Division of Population Health, Health Services Research & Primary Care, University of Manchester, Manchester, M13 9NT, UK; 10 Independent Advisory Panel on Deaths in Custody, London, SW1H 9AJ, UK

**Keywords:** Drugs and medicines, primary care, healthcare-associated harm, patient safety, medical management, prison medicine

## Abstract

**Objectives:**

Prisoners use healthcare services three times more frequently than the general population with poorer health outcomes. Their distinct healthcare needs often pose challenges to safe healthcare provision. This study aimed to characterise patient safety incidents reported in prisons to guide practice improvement and identify health policy priorities.

**Design:** We carried out an exploratory multi-method analysis of anonymised safety incidents from prisons.

**Setting:**

Safety incidents had been reported to the National Reporting and Learning System by prisons in England between April 2018 and March 2019.

**Participants:**

Reports were reviewed to identify any unintended or unexpected incident(s) which could have, or did, lead to harm for prisoners receiving healthcare.

**Main outcome measures:**

Free-text descriptions were examined to identify the type and nature of safety incidents, their outcomes and harm severity. Analysis was contextualised with subject experts through structured workshops to explain relationships between the most common incidents and contributory factors.

**Results:**

Of 4112 reports, the most frequently observed incidents were medication-related (n = 1167, 33%), specifically whilst administering medications (n = 626, 54%). Next, were access-related (n = 559,15%), inclusive of delays in patients accessing healthcare professionals (n = 236, 42%) and managing medical appointments (n =  171, 31%). The workshops contextualised incidents involving contributing factors (n = 1529, 28%) into three key themes, namely healthcare access, continuity of care and the balance between prison and healthcare priorities.

**Conclusions:**

This study highlights the importance of improving medication safety and access to healthcare services for prisoners. We recommend staffing level reviews to ensure healthcare appointments are attended, and to review procedures for handling missed appointments, communication during patient transfers and medication prescribing.

## Introduction

Improvement in prison healthcare safety is needed.^1^ There are approximately 80,000 people within prisons in England^2^ and this number is increasing.^3^ Prisoners have poorer health outcomes than the general population, with significant mental and physical healthcare needs.^4^^,^^5^ They are an ageing cohort with a high prevalence of chronic disease and pose increased demands on healthcare services.^3^^,^^6^ Prisoners use healthcare services three times more frequently than the general population^7^ and have distinctive health requirements that can be challenging to deliver. Healthcare priorities are often overshadowed by a prison’s main objective of securing detained individuals, creating ‘a twilight zone between criminal justice and health systems’,^8^ which risks neither adequately considering prisoner needs and their collective responsibility of care. Continuity of care is interrupted by a high turnover of prisoners, with more than 50% of prisoners incarcerated for fewer than six months^2^^,^^9^ and this causes them to be vulnerable to harm.

Prison healthcare delivery is summarised in supplementary Appendix 1 and despite difficulties in healthcare service provision, prisoners in Europe are legally entitled to healthcare that is comparable to the general population.^10^ Referred to as ‘equivalence’, care must be consistent in range and quality with what a patient would receive in a community setting.^11^ However, recent studies suggest that healthcare standards are not met, with prisoners attending fewer outpatient appointments (75% of appointments cancelled on the day), frequently attending hospital due to injury and poisoning, and emergency admissions with avoidable conditions such as diabetic ketoacidosis.^12^

Time within prisons could represent a period of stability to address prisoner health and wellbeing,^13^ although transitions (prison-to-prison, community-to-prison and vice versa) involved represent a period of risk to the prisoner and should be a target for care improvement.^14^ While issues compromising the quality and safety of prison healthcare are widely acknowledged, there is little research into understanding the nature and breadth of the problems and how this can be improved, to mitigate unsafe care for prisoners.

## Aim

We sought to characterise the type and nature of commonly occurring patient safety incidents within prisons and identify opportunities to improve existing healthcare systems within secure environments.

## Methods

We carried out a retrospective, multi-method analysis to characterise and explore the nature of prison-related patient safety incidents reported to the National Reporting and Learning System (NRLS),^15^ a central database in England and Wales (now the Learn from patient safety events (LFPSE) service). The National Health Service’s (NHS) definition of a patient safety incident, which is adhered to throughout our work, is ‘any unintended or unexpected incident which could have, or did, lead to harm for one or more patients receiving healthcare’.^16^

For healthcare-related incidents, prisons report in accordance with each healthcare provider’s protocol, which can include sharing incidents via management committees and with commissioners, and submit reports to the NRLS.^17^^,^^18^ Each report can contain structured information about the incident, including the location, as well as free text describing what happened, contributory factors and actions to prevent reoccurrence.

## Study population

Reports from the NRLS database were extracted on 24 September 2019, inclusive of incidents occurring from 1 April 2018 to 31 March 2019. This was an incident-level dataset, capturing incidents reported by English organisations. Reports were included that occurred in a ‘prison or remand centre’.

## Sample characterisation

We analysed patient safety incidents reported to the NRLS from prisons in England over a 12-month period.

The free text of each incident report was reviewed to ensure that they met the inclusion criteria below. Narratives were categorised using a multi-axial classification system developed by the PatIent SAfety (PISA) group at Cardiff University.^19^ Codes were applied systematically and chronologically, adhering to the ‘Recursive Model of Incident Analysis’^20^ (examples in supplementary Appendices 2 and 3).

## Report screening and inclusion criteria

Reports were included if they met the following criteria to allow descriptive analysis:

(1) Contained sufficient information to determine what happened.
(2)  Met the definition of a patient safety incident.^16^

All reports that met an additional third criterion were discussed at stakeholder workshops:

(3) Contained information to determine the aetiology (contributory factors) of the incident.

## Report coding

Incident reports were reviewed by trained coders (IJM and KD) and classified to describe incident type(s), contributory incident(s), contributing factor(s), harm outcome(s) and harm severity (see supplementary Appendix 4 for definitions), with codes assigned to reflect explicit content identified within the reports. No inferences or assumptions were made by the clinician coders. To ensure concordance between the coders, 20% of the reports were double-coded and kappa inter-rater reliability statistics were calculated. Each coding framework has been empirically developed in-house from the analysis of over 70,000 patient safety incident reports from the primary and community care context.^19^ The frameworks are collectively known as the PISA Classification System, which is aligned to the World Health Organization (WHO) International Classification for Patient Safety.^21^ Throughout coding, weekly team meetings took place to discuss the incident reports, emerging themes and any coding discordance.

An exploratory descriptive analysis of coded data was carried out to produce quantitative summaries and coded data was cross tabulated to identify the most frequently occurring incident types, contributory factors and outcomes, and to explore the semantic relationships between them.^22^

## Expert group workshops

Reports that met all three inclusion criteria were grouped by incident category. These reports were discussed and contextualised with subject experts and stakeholders through structured study team workshops, to examine and explain relationships between safety incidents and contributory factors. Our study team included experts from secure environments, pharmacy, sociology, criminology and lay people. Presentations were given to familiarise the group with the descriptive analysis of coded data. The workshops aimed to review, discuss hypotheses, highlight emerging themes and consider where recommendations could be made to improve healthcare and potentially avoid future similar incidents. To gain a deeper insight of the descriptive analysis, reports were re-reviewed, gathering expert perspectives and interpretations about emerging patterns. With permission from study team members, workshops were recorded and transcribed verbatim (IJM, reviewed by KD).

Throughout the study, in addition to six-weekly critical review and guidance from our Avoidable Harm study team,^23^ we presented our findings to the study Stakeholder Advisory Group (SAG), prison experts, NHS England and NHS Improvement and a Service User Group (previous detainees). This iterative process helped refine our understanding of findings and recommendations.

## Results

**Figure 1.**
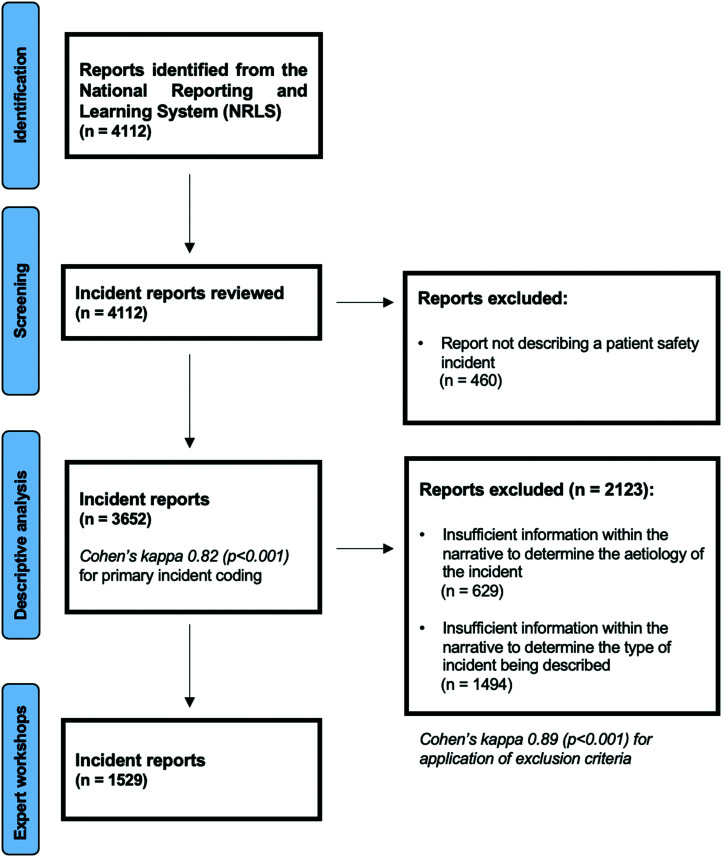
Summary of sample formation.

We screened 4112 patient safety incident reports (Figure 1). Following screening, 3652/4112 (89%) reports underwent descriptive analysis and 1529/3652 (42%) were discussed at the expert workshops.

Two trained coders (IJM and KD) screened and coded the reports. To assess concordance between reviews, Kappa inter-rater reliability statistics were calculated based on application of the exclusion criteria during screening (*k* = 0.89) and the allocation of primary incident type during coding (*k* = 0.82).

A total of 2982 (82%) reports contained one or more identifiable contributory factors; most commonly: patient behaviour (*n* = 810) contributed to an unsafe outcome, or issues relating to protocols, standards or guidance for enabling optimal care delivery were identified (*n* = 415).

Of the included reports for descriptive analysis (*n* = 3652), harm outcomes were explicit in 2778 reports (76%) and the most reported outcome was self-harm (*n* = 1095, 29%), followed by delays in management, assessment or treatment (*n* = 608, 17%). With the majority reported as no or low harm to patients, 2% resulted in serious harm or death.

The primary incident categories and top incident types included within the descriptive analysis can be seen in Table 1.

**Table 1.** Primary patient safety incident categories.

Primary incident category	*n* (% of incident reports included within descriptive analysis)	Top incident types within the category	*n* (% of incident category)
Grouped category (e.g. patient injury, falls, self-harm)	1363 (37)	Self-harm	1019 (75)
		Fall	93 (7)
Related to medications and vaccines	1167 (32)	Errors administering medication	149 (13)
		Errors dispensing medication	145 (12)
Administrative issue	559 (15)	Delay in accessing healthcare professionals	236 (42)
		Errors in managing appointments for healthcare	171 (31)
Related to diagnosis and assessment	132 (4)	Errors in the process of discharge planning	55 (42)
		Delayed assessment for care	23 (17)
Related to medical records	127 (3)	Errors in documentation/availability of medical records	77 (61)
		Medical records not up to date/complete	20 (16)
Related to equipment	85 (2.32)	Failure of equipment	27 (32)
		Lost equipment	13 (15)
Related to treatment	72 (1.97)	Insufficient treatment/care	35 (49)
		Errors in the treatment decision process	10 (14)
Incidents not found in other categories, e.g. related to transportation	71 (1.94)	Errors in transport logistics	47 (66)
		Errors in the professional conduct of healthcare professionals	8 (11)
Related to clinical investigations	32 (0.87)	Errors in the process of obtaining or processing a laboratory specimen	8 (25)
		Errors in the process of reporting laboratory investigations	6 (19)
Related to clinical referrals	26 (0.7)	Referral not performed when indicated	8 (31)
		Errors in transfer of patient notes	4 (15)
Related to communication within healthcare	18 (0.5)	Errors in communication between healthcare professionals and other professionals, e.g. prison staff	7 (39)
		Errors in communication between healthcare professionals	5 (28)
Total	3652		

## Medication-related incidents

Medication-related incidents were the most frequently reported incident category (*n* = 1167/3652, 32%). Incidents involving the process of administering medications, for example, where medication could not be administered (*n* = 142, 23%) as seen in Table 2, Example 1, were frequently observed (*n* = 626/1167). Reports discussed within the expert workshops are further stratified in supplementary Appendix 5.

**Table 2.** Example incident reports.

Example 1	‘Patient ran out of anti-epileptics and informed nursing staff. Prescription was not in place to be renewed. Prescription chased and done. Faxed to pharmacy, but no stock available for that day. Medication not received until two days later, patient missed medication and suffered three seizures …’
Example 2	‘[Name of electronic medical record system] failure. Staff were unable to access medical records, prescriptions, or duty ledger. Delay in medication at treatment hatches and Methadone dispensing. Delay in accessing patients records and completing ledger appointments including emergency response ledger and daily observations’.
Example 3	‘Prisoner has a double prescription – one from their previous establishment and one from the GP (General Practitioner) at current area. On [electronic record] we cannot see that one is from previous establishment, therefore there is a risk of giving a double dose. Nurses informed to be aware of this risk and GP sent a task to cancel extra prescription’.
Example 4	‘Patient restrained as they refused to go back to their cell. Resulted in rupture of prisoner’s finger and severe wound. Assessed by medical staff as needing urgent transfer to A&E. On discussion with prison management, overrode GP decision to refer to A&E based on short staffing’.
Example 5	‘Patient awaiting USC (Urgent Suspected Cancer) referral for an endoscopy. Handover was not given to staff/patient that fasting was required. This was due to security lockdown and as such patient not fasted and unable to attend assessment’.
Example 6	‘Prisoner came in as new admission. Mentioned at screening that she had a lump in her breast. Urine screen negative so not seen by reception GP. Nurse sent task requesting routine nurse appointment for review. Appointment wasn't booked until *some time* later. Assessed by nurse who requested GP appointment. Seen by GP next day who assessed. Concerning symptoms requiring USC referral to Breast Clinic. Unfortunately, prisoner was released with both a weekend and bank holiday in between so not enough time for referral to be processed. Prisoner was released to a safe house and did not know address, neither does she have a registered GP in this area, thus making it difficult to pass on relevant details for this to be followed up in the community. If this had been flagged up as urgent for GP at reception, it may have been possible to get her seen in Breast Clinic before she was released’.
Example 7	‘Prisoner’s cellmate pressed call bell that was answered by prison officer. Told that prisoner feeling unwell, slurring speech and facial weakness and stated he thought he was having a stroke. Cellmate asked if prisoner could be seen by a nurse, but this was not relayed to nursing staff. Prisoner was seen the next day and sent to the hospital with a suspected stroke’
Example 8	‘Urgent first appointment for treatment of newly diagnosed lymphoma. Categorised as “red” (urgent, life threatening and do not cancel) by a senior doctor. Security staff unilaterally cancelled and rebooked this appointment for a later date. This will delay cancer treatment’
Example 9	‘Recommendation made by consultant psychiatrist that patient requires inpatient admission on prison healthcare unit and urgent referral to hospital. Decision on this has been overruled by the prison due to current level of unlock required and risk to others. This poses direct impact on the ability to clinically assess the patient and having access to mental health professionals. Patient is not suitably located, and referral has not been sent’.
Example 10	‘Tried to access a palliative care patient residing in the inpatient department. However, I was unable to see him as a non-healthcare prisoner was on exercise and the patient was locked behind his door for safety reasons, even though the dying patient has an open-door policy in place’.

The most frequently reported contributory factor within medication-related incidents was mistakes made by staff members (*n* = 299), for example, arising where patients had similar names (*n* = 59). Further information regarding contributory factors can be found within supplementary Appendix 6.

One-fifth of all reported medication-related incidents were detected and/or mitigated by staff (*n* = 197) or patients (*n* = 32) (Table 2, Example 3). Within incidents that caused harm to the patient, over half resulted in patients receiving the incorrect treatment (*n* = 534) or missed doses of medication (*n* = 250). Missed medication was usually not named within the report or involved the following medications: antibiotics, anti-epileptics and chemotherapy drugs (*n* = 44).

## Incidents relating to the ‘ability to access healthcare professionals’

Delays or the inability to access healthcare included challenges to access healthcare professionals (*n* = 236, 57%) and appointments at external hospitals (*n* = 171, 43%). The most frequently reported contributory factors included insufficient provision of prison and healthcare staff (*n* = 154) and difficulties with access due to security-related barriers; for example, when prison wings were on lockdown (*n* = 148) (Table 2, Examples 4 and 5).

Difficulties in accessing healthcare led to delays in the assessment, management and treatment of patients (*n* = 325), including chest pain, alcohol withdrawal and signs of a stroke (Table 2, Examples 7 and 8). Most of these incidents were reported to have caused no harm (*n* = 372) to patients. Where incidents resulted in patient harm, there was a further impact on staff workload (*n* = 174), with increased administrative work (*n* = 24), for example, additional training and increased pressure on staff to carry out further work.

For a detailed analysis of incidents related to diagnosis and assessment, clinical records, transitions of care, and the most reported outcome of self-harm, see supplementary Appendix 7.

## Key themes from workshops

An analysis of expert and stakeholder discussion with the coded data generated the following key themes.

### Equitable healthcare access

Issues with prisoners accessing healthcare were observed across all incident categories, including the prisoner’s inability to access healthcare professionals, with security constraints and lockdowns hampering attendance of internal appointments (Table 2, Examples 7 and 8). Medication delivery was affected by security lockdowns and prisoners were unable to approach medication hatches, resulting in medication being missed. External appointments were regularly affected by an insufficient number of prison officer escorts.

### Continuity of care

Continuity of care was variable, especially for medication delivery processes. Incidents occurred during prescribing, dispensing and administration. Times of transition disrupted continuity of care (Table 2, Example 6), and staff/patients often mitigated potentially harmful outcomes (Table 2, Example 2).

### Balance between healthcare and prison priorities

Competing demands of security and healthcare was identified across all incident categories. This disparity was often underlined by poor escalation protocols and inefficient communication between healthcare services and prisons (Table 2, Examples 7, 9 and 10). This was often seen during the delivery of care to vulnerable patients related to their mental health and managing acutely unwell patients.

## Discussion

### Principal findings

This is the first national-level study to characterise the nature of prison-related patient safety incidents. While our results highlight that medication-related incidents are frequently reported, particularly in relation to their administration, these reports often resulted in low harm incidents, with staff, detecting and mitigating poorer outcomes for patients.

Reduced access to healthcare was seen across the continuum of the prisoner’s healthcare journey, which led to delays in care and treatment. Our results highlight risk to patient safety within the secure environment, particularly in relation to high-risk clinical presentations and acutely unwell patients. Access to healthcare within prisons highlights conflict between security regimes and healthcare priorities, correlating with concerns of prisoners reported from Australia previously, where medication management and healthcare service access were also clear priorities for policymakers and further research.^24^

## Contextualisation with current literature

### Community and prison settings

Prisons are ‘part of society – therefore prisons are crucial for sustaining and advancing public health’.^8^ It is therefore pertinent to draw comparisons between the existing literature from the community context and studies specific to secure environments.

Compared with a national study that reviewed over 13,500 incident reports submitted to the NRLS from primary care within England and Wales,^25^ our study found similar issues around poor communication between teams, medical referrals, the discharge of patients and transfer of information. Most incidents involved clinician decision-making issues and delayed management or mismanagement. Outside of the prison environment this concerned a failure to recognise signs of clinical deterioration; however, within prisons, clinical deterioration was often identified, but not dealt with appropriately or poorly escalated due to security constraints.

## Medication

Medication-related incidents were frequently reported, corresponding with incidents seen in the community.^25^

Access to medications was a key theme within prisons, with missed medication accounting for 10% of all medication outcomes. This correlates to a recent report, ‘Deaths in Prison’,^26^ that noted delays or poor access to medication can cause significant impact on physical and mental health. Despite missed critical medication being a key target to be addressed in the Royal College of General Practitioners guidance about prescribing in prisons,^27^ we identified that access to medication remains an issue, even for critical drugs where timeliness of receipt is essential. Details describing who prescribes medication within prisons and the challenges involved are provided in supplementary Appendix 1.

Unlike community-based studies, where prescribing and dispensing errors are more commonly reported,^25^ within prisons we found that incidents related to the administration of medication was reported most frequently. This could reflect prisons relying on staff to distribute and often administer medications^27^ – most like that present within nursing homes or secondary care, and not the autonomous administration within the community.

A mixed-methods study from 2021, involving synthesis of prescribing safety indicators, a literature review and nominal group discussion at two English prisons identified similar issues.^28^ Notably, prescribing practices that could cause harm were clustered around several high-risk medications, including selective serotonin re-uptake inhibitors and anti-psychotics, which echoes the medication classes described in reports included in our study. Building on this, we shed light on issues involving poor medication access, including delays in receiving these medications.

## Healthcare access

The British House of Commons Health and Social Care Committee in the United Kingdom (UK) recognised that the Government is failing prisoners within England, and they have breached their duty of care with regard to unsafe premises and the inability of prisoners to access appropriate healthcare.^29^ We have highlighted the high frequency of reports filed by prison-based healthcare professionals where prisoners have not been able to access healthcare professionals and medical appointments.

Other studies have described similar concerns, stating ∼40% of prisoners miss their external hospital appointments.^12^ Our findings corroborate and strengthen their recommendations that to improve prisoner’s access to healthcare, communication must be strengthened between the prison and healthcare staff within prisons, as well as priority planning to ensure sufficient numbers of prison escorts are available when needed.

With the COVID-19 pandemic as a catalyst, the volume of ‘telemedicine’ and remote consultations has increased across all healthcare settings. Remote access appointments could remove many of the physical and security constraints we have highlighted and could improve prisoners’ access to healthcare. The Nuffield Trust^12^ in the UK have made recommendations about remote consulting, finding that the number of remote consultations has increased, particularly in specialties such as Trauma and Orthopaedics, which has gone some way to improving access to specialist services for prisoners.

## Prison security

Security-related constraints pose unique challenges for healthcare delivery in prisons. Previous studies have highlighted the impact on internal medical clinic attendance due to security lockdowns, for example after incidents within prison wings,^30^ and recognise the need to consider constraints proactively and regularly on care delivery and care access. This lack of access is corroborated in a summary of prisoners’ views from across the UK,^29^ which found that up to 75% of all prisoners feel that healthcare services were unattainable.

With a reduced volume of prison staff,^3^ the requirement for escorts, planning and resourcing to improve access needs to be prioritised. Currently, the requirement for a prisoner to attend an appointment is assessed according to urgency. This assessment needs to become objective, outcomes transparent, and where attendance is not possible, robust communication established to ensure that the patient is not lost to follow-up.

## Strengths and limitations

This is the first multi-method analysis of safety incident reports from secure environments in England. We used an established method of recursive coding to gain a higher-level understanding of the breadth of the reports. We were meticulous in the application of our methods with high inter-rater reliability and audit trails of coding decisions and analysis.

It is widely acknowledged that incident reports have limitations for understanding patient safety. Typically, learning can be hindered by reporter and selection bias and the variation in report quality. A significant number of reports were excluded from the expert workshops and further review due to insufficient information to decipher what happened to the patient or due to including information regarding the patient outcome only, for example self-harm. The breakdown of reports by NRLS coding can be found in supplementary Appendix 8. Our conclusions and recommendations can only be derived from a section of the reports reviewed. In addition, given the total number of prisoners in England and Wales,^2^ the 4112 reports could reflect underreporting, though they are comparable to that seen in primary care.^25^

## Recommendations that may resolve some of the issues identified by the study

1. Enabling prisoner access to healthcare.
  - Staff rota review to ensure sufficient healthcare staff and prison officers for clinic and other appointments to take place.
  - Sufficient training and policies required, especially for agency staff or those unfamiliar to prison settings, with strategies for avoiding missed healthcare appointments, especially related to vulnerable and critically unwell patients.
  - Prison layouts should be reviewed to ensure that access to patients is optimised to ensure timely access for healthcare professionals (internal and external) to reach those in greatest need during lockdowns.
2. Optimising management of external healthcare appointments.
  - Escalation policies are needed for missed appointments to reduce their frequency and to ensure, if they do occur, correct action has been taken to ensure the patient is not lost to follow up.
  - Appropriate planning for escorts to allow for attendance at healthcare appointments.
  - Increased adoption of telemedicine and remote consulting, when possible, to reduce the requirement of prisoners needing to leave the premises.
  - Increased provision of in-reach clinics, where a specialist attends the prison, to reduce the volume of escorts required.
3. Improving the process and procedures around transfer of care.
  - Improved communication during prison transfers, this might include the reliable use of protocols and standard handover sheets to arrive with prisoners to ensure continuity of care.
  - Clear policies on the safe transfer of medication within prison and improved continuity of care during transfers between prisons or from prison to the community particularly for prescribing and dispensing of medication.
  - Appropriate discharge planning and communication with the community.
4. Ensuring improved quality of data from reporting.
  - Additional guidance to support storytelling whilst completing patient safety incident reports within secure environments is needed; consider adopting the ‘Situation, Background, Assessment and Recommendation’ (SBAR) framework for structuring narrative reporting.
  - Provide staff educational modules to help improve the quality of all incident reports.

## Conclusion

Our national-level analysis of patient safety incidents reported from secure settings highlights that substantive improvements are needed to improve patient safety in prison-based healthcare in the UK and these implications have international relevance. Prisons are a unique context for healthcare delivery where a hybrid model of both primary and secondary care input is in place. As such, our study identifies priorities for safety improvement in this context, notably in relation to the interplay between healthcare and prison security. It is vital that prisons enable timely access to healthcare services and optimise processes to mitigate medication-related harm.

## Supplemental Material

sj-pdf-1-jrs-10.1177_01410768231166138 - Supplemental material for Patient safety in prisons: a multi-method analysis of reported incidents in England Click here for additional data file. Supplemental material, sj-pdf-1-jrs-10.1177_01410768231166138 for Patient safety in prisons: a multi-method analysis of reported incidents in England by Isobel J McFadzean, Kate Davies, Thomas Purchase, Adrian Edwards, Stuart Hellard, Darren M Ashcroft, Anthony J Avery, Sandra Flynn, Tom Hewson, Melanie Jordan, Richard Keers, Maria Panagioti, Verity Wainwright, Florian Walter, Jenny Shaw and Andrew Carson-Stevens in Journal of the Royal Society of Medicine

## Declarations

### Guarantor

ACS.

### Availability of data and materials

Incident reports used for the study are available, by application, from NHS England only. The IDs of all reports used for the study can be provided upon reasonable request. Due to the risk of identifying individual participants, the workshop transcripts will not be made available.

### Provenance

Not commissioned; peer reviewed by Peter Ross, Charles Vincent and Julie Morris.
