# Supplementary material for: Patient safety in prisons: a multi-method analysis of reported incidents in England
Source: J R Soc Med. 2023 May 17;116(7):236–45. doi: 10.1177/01410768231166138 (PMC10387805; doi:10.1177/01410768231166138)
Supplement: sj-pdf-1-jrs-10.1177_01410768231166138 - Supplemental material for Patient safety in prisons: a multi-method analysis of reported incidents in England [file sj-pdf-1-jrs-10.1177_01410768231166138.pdf]

Supplementary materials

Patient safety in prisons: a multi-method analysis of reported incidents in England

Appendix contents

**Pages 2 - 5**	**Appendix 1.** Overview of how healthcare is delivered within prisons
**Page 6**	**Appendix 2**. Rules of the Recursive Model of Incident Analysis
**Pages 7**	**Appendix 3.** Hypothetical vignette example to illustrate coding processes
**Page 8**	**Appendix 4.** Definitions of key terminology
**Pages 9 - 11**	**Appendix 5.** Medication incidents reviewed at expert workshops.
**Page 12 -13**	**Appendix 6.** Summary of the contributory factors associated with medication-related incidents
**Pages 14 - 15**	**Appendix 7.** Summary of findings for incidents related to diagnosis and assessment, clinical records, transitions of care, and self-harm
**Pages 16 - 17**	**Appendix 8** Summary of NRLS coded incident categories

Appendix 1. Overview of how healthcare is delivered within prisons

In 2006, the Department of Health took over the provision of healthcare in prisons in England and Wales from the Prison Medical Service with a view to support equivalence of primary care services[1, 2]. This means prisons must be provided with the "staff, resources and facilities of at least the same standard as those available in the community" RCGP [2]. Following the restructuring of the NHS, from April 2013, NHS England Health and Justice have been responsible for the commissioning of all healthcare services for prisoners [3] and contracts for prison healthcare delivery can be issued to both NHS and private providers [4]. In addition to equivalence, NHS England's mandate is to ensure continuity of care across the criminal justice pathway [5].

Legislative changes in 2022 mean that integrated care systems (ICS) (to integrate care across local system partners) have been developed in local areas in England. However, the responsibility for prison healthcare has not yet been delegated to ICSs and remains centrally coordinated [4]. This is likely due to the high levels of physical and mental health need amongst the prison population [6] and the complexity of delivering healthcare services in prison [4].

Healthcare provision and delivery across the prison estate varies, but all prisons undertake a health screening of new prisoners to establish their healthcare needs, and there is a process for prisoners to request and book healthcare appointments. Primary care is available in all prisons with some healthcare services available on site, whilst others have dedicated healthcare wings with inpatient beds (the size of these wings also varies across the estate).

While there are core health and medical staff who work within prisons, to varying degrees there is also reliance on agency nurses and locum General Practitioners (GPs). Healthcare practitioners may also visit to deliver healthcare clinics (for example, opticians, podiatrists, and physiotherapists) but the quality of joint working across the different providers for things like mental health, dentistry and so on is variable [7].

Prisoners have less opportunities for self-care due to the inherent security of the environment [6]; for example communication about, and attendance at, clinic appointments. When outside hospital appointments are required, these will always require prison officer escort due to the risk of a prisoner absconding and consequently, staff shortages and emergency security issues can lead to cancellations of appointments, often immediately prior to the time they are due to be seen. Prison officers and allied security staff may also play a significant role within the escalation of healthcare needs, occasionally being first to respond in clinical emergencies and often relay clinical information to doctors and healthcare professionals (HCPs), and can be described as an unwanted companion by some prisoners [8].

For a detailed discussion of prison healthcare in England, the following resource by the Nuffield Trust resource is available: https://www.nuffieldtrust.org.uk/resource/prison-health-care-in-england [9].

Within English prisons there are also several HCPs who may prescribe medications (including GPs, non-medical prescribers, Psychiatrists, and substance misuse specialists). These HCPs may not always be based within the prison, and may by employed by NHS or private organisations commissioned to provide health care services [10].

Prisoners are known to present with elevated rates of morbidity, including mental illness [11] and substance misuse [10, 12] and prescribing medication takes place in a setting with high-prisoner turnover, enduring risk of medication-trading and prisoner self-harm and where security heavily influences medicines management [10, 13, 14].

In order to deliver care for prisoners that is 'equivalent' to the general population, prescribers must adapt their practice and consider this complex prison environment and patient population, including the consequences of their decisions for both the prisoner and wider prison population [10, 13, 15]. The safety of prescribing in prisons is influenced by a number of challenges; for example those related to variable secure environment structures and governance, staffing levels and skills mix, patient factors (e.g. drug seeking behaviours, co-morbidities/polypharmacy) and a lack of alternative options to medication [10, 13, 15].

Our findings concerning the importance of medication-related safety challenges in prisons are supported elsewhere in the literature; for example, in psychotropic prescribing where elevated rates of use compared to the general population accompany issues such as polypharmacy, poor documentation, high dose prescribing and insufficient monitoring [12, 16, 17] This highlights a need for further research involving multidisciplinary team approaches that recognises and addresses these challenges to improve patient care.

References

1. Executive, National .Health .Service., *The future organisation of prison health care*. 1999.
2. Royal College of General Practitioners, *Equivalance of care in Secure Environments* 2018.
3. National Health Service . *Health and Justice* 2021; Available from: https://www.england.nhs.uk/commissioning/health-just/.

4. Edge, C., et al., *Integration, population commissioning and prison health and well-being–an exploration of benefits and challenges through the study of telemedicine.* Journal of Integrated Care, 2022. **30**(5): p. 108-124.
5. Ministry OfJustice, *National Partnership agreement for Prison Healthcare in England* 2018.
6. Bradshaw, R., et al., *The health of prisoners: summary of NICE guidance.* BMJ, 2017. **356**.
7. Hayton, P. and J. Boyington, *Prisons and health reforms in England and Wales.* American Journal of Public Health, 2006. **96**(10): p. 1730-1733.
8. Edge, C., et al., *Secondary care clinicians and staff have a key role in delivering equivalence of care for prisoners: A qualitative study of prisoners' experiences.* EClinicalMedicine, 2020. **24**: p. 100416-100416.
9. Hutchings, R.a.D., M, *How prison health care in England works*. 2021.
10. Magola-Makina, E., et al., *Exploring the challenges to safer prescribing and medication monitoring in prisons: A qualitative study with health care staff.* Plos one, 2022. **17**(11): p. e0275907.
11. Hassan, L., et al., *Accounting for psychotropic medication changes in prisons: Patient and doctor perspectives.* Qualitative Health Research, 2015. **25**(7): p. 954-965.
12. Hassan, L., et al., *Prevalence and appropriateness of psychotropic medication prescribing in a nationally representative cross-sectional survey of male and female prisoners in England.* BMC psychiatry, 2016. **16**(1): p. 1-10.
13. Royal College of General Practitioners. *Safer Prescribing in Prisons: Guidance for clinicians* 2019; Available from: https://bulger.co.uk/prison/RCGPsaferprescribinginprisons2019..pdf.
14. Hampton, S., et al., *Prescribing for people in custody.* Australian Prescriber, 2015. **38**(5): p. 160.
15. Bartlett, A., et al., *Prison prescribing practice: practitioners' perspectives on why prison is different.* International Journal of Clinical Practice, 2014. **68**(4): p. 413-417.
16. Griffiths, E.V., J. Willis, and M.J. Spark, *A systematic review of psychotropic drug prescribing for prisoners.* Australian & New Zealand Journal of Psychiatry, 2012. **46**(5): p. 407-421.
17. Abuzour, A.S., et al., *Implementing prescribing safety indicators in prisons: A mixed methods study.* British Journal of Clinical Pharmacology, 2022. **88**(4): p. 1866-1884.

Appendix 2. Rules of the Australian Recursive Model of Incident Analysis

**Incident Analysis Rules**	**Rule Example**
**1. An incident has a set of contributory factors and / or contributory incidents**	Missed diagnosis (incident) because the doctor did not adequately examine the patient (contributory incident) and the doctor had inadequate knowledge (contributory factor)
**2. An incident can contribute to another incident**	Missed diagnosis (contributory incident) resulted in a patient not receiving a timely referral to the hospital (primary incident)
**3. Contributory factors cannot be incidents in their own right**	A mistake (contributory factor *not* an incident) resulting in the wrong prescribed medication dose (primary incident)
**4. An incident has a set of outcomes**	Wrong prescribed medication dose (primary incident) resulting in a medication overdose and hospital admission (outcomes)
**5. An incident can be an outcome of another incident**	Records not up to date (contributory incident) resulting in the wrong prescribed medication (primary incident and outcome)
**6. Some outcomes cannot be incidents in their own right**	Admission to hospital (outcome) following the wrong prescribed medication (primary incident)
**7. An outcome of an incident could be a contributory incident to another incident**	Communication incident between care providers (contributory incident) resulting in records not being up to date (contributory incident and outcome), resulting in a referral incident (primary incident)
**8. An incident can be designated the primary incident type – the incident proximal to the descriptive patient outcome**	Communication incident (incident) leading to inaccurate records (incident), leading to the wrong prescribed medication (primary incident type)
**9. The outcome of a primary incident cannot be an incident**	Admission to hospital (outcome) following the wrong prescribed medication dose (primary incident type)

Appendix 3. Hypothetical vignette example to illustrate coding processes

This following vignette is an example report, created from an amalgamation of different anonymized incident reports from prisons.

"Patient missed an appointment yesterday – they were supposed to be seen in hospital for consideration of surgery. Officer forgot to hand over a message about the appointment and there weren't enough prison officers for an escort today.

I spoke to [staff member] in the outpatient department today, but because we hadn't informed them that [patient name] would not be attending yesterday, they have discharged them. I've also been told that they will now need to be re-referred by the GP and there is a significant waiting list – which will result in a delay in having the surgery. There is currently no way to escalate this when prisoners cannot attend their appointments, and this is not the first event that I've had to report this week..."

Recursive model based on Australian Federation patient safety organisation.

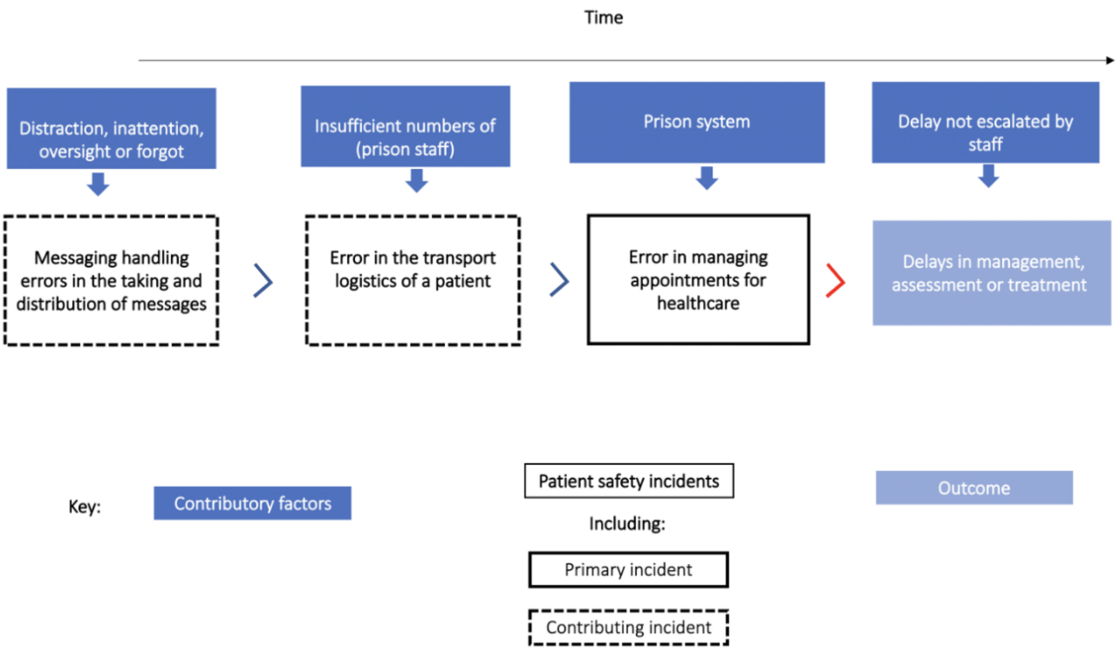

The vignette is de-constructed into a series of codes. Appendix 4 further details the definitions used, and the 'primary incident' is related to the management of healthcare appointments due to it specifically detailing issues regarding a prisoner's outpatient appointment, and within the vignette there is evidence of 'contributory incidents' related to transport and communication. In addition, 'contributory factors' are evident where the reporter explicitly states that an officer had forgotten to hand over the message, issues with staffing levels to act as an escort, and direct mention of the prison system. The 'outcome' for the patient would be a delay in management, due to the delay in subsequent surgery, following these issues.

Appendix 4. Definitions of key terminology

Term	Definition
**Patient safety incident**	Any unintended or unexpected incident which could have, or did, lead to harm for one or more patients receiving healthcare [16].
**Harm**	• None – patient outcome is not symptomatic, or no symptoms detected, and no treatment is required.<br>• Mild – patient outcome is symptomatic, symptoms are mild, loss of function or harm is minimal or intermediate but short term, and no or minimal intervention (e.g., extra observation, investigation, review, or minor treatment) is required.<br>• Moderate – patient outcome is symptomatic, requiring intervention (e.g., additional operative procedure; additional therapeutic treatment), an increased length of stay, or causing permanent or long-term harm or loss of function.<br>• Severe – patient outcome is symptomatic, requiring life-saving intervention or major surgical/medical intervention, shortening life expectancy, or causing major permanent or long-term harm or loss of function<br>• Death – on balance of probabilities, death was caused or brought forward in the short term by the incident [21].
**Primary incident**	The incident, usually an issue, complication, or lack of something perceived important during a task or process of care delivery, occurring prior to the outcome and is the incident most proximal to the identified patient outcome [21].
**Contributory incident**	An incident that is proximal to the outcome that has contributed to the primary incident [21].
**Contributory factor**	The circumstances, actions or influences which have played a part in the origin or development of an incident or to increase the risk of an incident [21].
**Outcome**	The impact upon a patient which is wholly or partially attributable to an incident [21].

Appendix 5. Medication incidents reviewed at expert workshops

Incident theme n (% of medication incidents)	Related primary incident	Total
**Administering medication 344 (44)**	Error in the process of administering medication to a patient	91
	Medication not administered	73
	Medication administered at incorrect dose (unspecified)	65
	Incorrect medication administered	41
	Medication administered to the incorrect patient	32
	Overdose administered	13
	Medication administered at the incorrect time	8
	Underdose administered	6
	Contraindicated medication administered	5
	Out of date medication administered	3
	Wrong number of doses administered	2
	Wrong formulation administered	2
	Medication stopped in primary/secondary care but administered still	2
	Medication administered via the incorrect route	1
**Dispensing Medication 186 (24)**	Dispensing medication orders incorrectly	124
	Medication dispensed at incorrect dose (unspecified)	12
	Medication dispensed to incorrect patient	11
	Medication not dispensed	11
	Incorrect medication dispensed	9
	Wrong label on medication	6
	Medication that had been stopped still dispensed to patient	4

	Wrong number of doses dispensed to patient (unspecified)	2
	Overdose dispensed to patient	2
	Medication dispensed for the incorrect route	1
	Wrong formulation dispensed	1
	Medication dispensed that is contraindicated for the patient	1
	Dispensed out of date medication	1
	Incorrect quantity of medication suppled (unspecified)	1
**Prescribing medication 145 (19)**	Duplicate prescription	60
	Wrong medication prescribed	59
	Prescription inaccurate or incomplete	12
	Medication prescribed for the incorrect time	4
	Wrong number of doses prescribed (unspecified)	2
	Medication has been stopped from being prescribed	2
	Wrong dose prescribed (unspecified)	2
	Overdose prescribed	1
	Illegible prescription	1
	Contraindicated medication prescribed	1
	Prescription lost	1
**'Other" – incidents found outside of the above themes 103 (13)**	Medication not taken by patient	26
	Medication stored incorrectly	19
	Medication not commenced in a timely fashion	14
	Medication not available	10
	Related to medication	5
	No medication arranged when appropriate	4
	Lack of medication monitoring	4
	Illegal supply of medication	3
	Patient overdose (unspecified)	3
	Formulation issue (unspecified)	2
	Vaccine related	2

	Medication lost by healthcare professional (unspecified)	2
	Insufficient medication (unspecified)	1
	Error in the decision - making process related to medication (unspecified)	1
	Wrong medication (unspecified)	1
	Unsuitable medication taken by patient	1
	Medication monitoring error	1
	Delay in receiving a vaccine	1
	Allergic reaction developed	1
	Medication lost	1
	Medication took wrong route	1
	**Total**	**778**

Appendix 6. Summary of the contributory factors associated with medication-related incidents

Top contributory factors<br>n	Frequency of top contributory factors within different incident categories<br>n	Frequency of the contributory factors found within incident types<br>n
**Mistake**<br>**n=299**<br><br>Examples:<br>• *Similar patient names (n=59)*<br>• *Distraction (n=28)*<br>• *Misread (n=12)*	Process of administering medications<br>82	Wrong dose administered<br>25
		Wrong medication administered<br>20
	Dispensing medication<br>62	Medication dispensing error<br>40
		Incorrect dose dispensed<br>6
	Prescribing medication<br>39	Prescribing medications error<br>22
		Wrong dose prescribed<br>6
**Failure to follow protocols**<br>**n= 271**<br><br>Examples<br>• *New protocol (n=5)*<br>• *Wrong professional carries out task (n=4)*	Administering medications<br>79	Administering medication error<br>20
		Wrong dose administered<br>14
	Dispensing<br>28	Medication dispensing error<br>15
		Wrong medication dispensed<br>5
	Other (e.g., medication storage)<br>20	Incorrect medication storage<br>16
		Medication unavailable<br>3
**Insufficient protocols, policies, or procedures**<br>**n=191,**<br><br>Examples<br>• *Repeat prescribing (n=28)*	Administering<br>39	Administering medications error<br>9
		Medication not administered<br>8
	Prescribing<br>56	Duplicate prescription<br>43
		Prescribing medications error

• *Dispensing protocols (n=19)*<br>• *Poor prescription design (n=6)*		11
	Dispensing<br>18	Medication dispensing error<br>9
		Wrong medication dispensed<br>2
**Working conditions n=153**<br><br>Examples:<br>• *Numbers of staff (n=74)*<br>• *Staff behaviour (n=27)*<br>• *Disagreement between teams (n=11)*	Administering<br>42	Administering medication error<br>18
		Medication not administered<br>16
	Prescribing<br>10	Prescribing medication error<br>6
		Duplicate prescription<br>4
	Dispensing<br>10	Medication dispensing error<br>6
		Medication not dispensed<br>2

Appendix 7. Summary of findings for incidents related to diagnosis and assessment, clinical records, transitions of care, and self-harm

Diagnosis and assessment-related incidents

Overall diagnosis and assessment-related incidents accounted for 4% of the total incidents (n=132), these included reports detailing the assessment process (n=45), diagnostic process (n=13) and discharge processes (n=57).

Incidents related to assessment processes accounted for 34% (n=45) of incidents in this category (Table 3, Example 6). These include delayed assessment (n=25), insufficient assessment (n=10) and incidents related to mental health assessments (n=7). The associated contributory incidents included insufficient staffing (n=8), issues with continuity of care (n=7) and difficulties due to the secure environment (n=5). These incidents caused delays in assessment, management, or treatment (n=24) and hospital admissions (n=6).

Incidents related to the diagnostic process (n=13) often led to delays in the assessment, management, and treatment of patients (n=5). They involved missed diagnoses (n=3), delayed diagnoses (n=3) and an incorrect diagnosis (n=1) including significant conditions such as deep vein thrombosis (DVT) (Table 3, Example 7). Insufficient protocols (n=4), mistakes (n=2) and the secure environment (n=2) were frequently reported contributory factors.

Discharge planning incidents accounted for 1.5% (n=57) of all incidents. They were most frequently associated with compromised continuity of care (n=31), inadequate protocols (n=19) and barriers arising from security-related requirements present within the prison at the time of the incidents (n=12). These incidents caused delayed discharge (n=12), unclear outcomes for the patient (n=11) and missed medication doses (n=8).

13% of the reports (n=17) described instances where patients symptoms did not appear to be appropriately escalated. Contributory factors included continuity of care (n=6), failure to follow protocols (n=5) and issues pertaining to the secure environment (Table 3, Example 8). As with issues with diagnosis, assessment and discharge, delays in management, assessment or treatment accounted for many of the outcomes (n=11), alongside staff outcomes (n=2).

Incidents related to clinical records

Incidents associated with clinical records accounted for 3% (n=127) of all incidents. From these reports, incorrect documentation (n=77) was the most frequently seen. Contributing to these incidents were failures to follow protocols (n=33), mistakes (n=16), and issues related to information technology (IT) (n=12). Overall, most outcomes associated with these incidents were unclear (n=25), alongside staff preventing harm (n=20), and legal implications (13) such as breaches of confidentiality.

Inaccurate, incomplete, or outdated records (n=47) were associated with failures to follow protocol (n=27), poor continuity of care (n=14) and tasks that were not completed (n=7).

These most frequently resulted in organisational inconvenience (n=22), such as increased documentation (n=2) and increased phone calls (n=2).

Incidents related to transitions of care

Overall, 11% (n=399) of incident reports occurred at times of transitions of care, both within the prison, between prisons and with external care providers e.g., community and secondary care. Of these, 197 reports described transitions as the primary incident. Where points of transition were contributory incidents (n=202), the most frequently reported associated primary incidents were medication related (n=136) (Table 3, Example 3), namely errors with dispensing (n=48) and with administering (n=41). Issues with accessing healthcare were also frequent, with errors in accessing healthcare professionals within the prison (n=51) and with accessing external appointments (n=88). The transfer of patient information was the most frequent primary incident related with care transition (n=85), followed by incidents related to discharge (n=57).

Contributing to these incidents were the lack of continuity of care (n=128), the secure environment (n=78) and failures to follow protocols (n=58). Incidents occurring at times of care transition resulted in delays to assessment, management, and treatment (n=176) as well as missed doses of medications (n=74). Around one tenth of the outcomes were mitigated by staff, and further harm prevented. Most incidents resulted in no harm (n=346), with 10% accounting for low harm, and 3% for moderate harm.

Incidents resulting in self-harm

Whilst the most frequently reported outcome for patients was self-harm (n=1,095), the quality of reports detailing these events was poor. Reports contained little information regarding the patient safety incident leading to, or resulting in, the episode of self-harm, and over a third of reports (n=336) contained no discernible contributory factors. Of those that did contain contributory factors, most were reported to be related to the patients themselves (n= 685) for example, their worsening mental health within the prison, as well as decision-making processes (n=560) playing an integral role, for example, planning and taking an overdose of in-possession medication. Reports often highlighted the clinical consequences of self-harming, for example additional monitoring by healthcare staff, further investigations (blood tests following overdoses) or treatment (suturing, or admission to hospital) (n= 420).

Appendix 8 Summary of NRLS coded incident categories

Incident category	Total number of reports	Number of reports included for expert workshop discussion	Number of reports excluded from expert workshop discussion
**Self-harming behaviour**	1319	40	1279
**Medication**	1062	767	295
**Infrastructure (including staffing, facilities, environment)**	268	73	195
**Access, admission, transfer, discharge (including missing patient)**	268	183	85
**Treatment, procedure**	196	136	60
**Patient accident**	190	15	175
**Disruptive, aggressive behaviour (includes patient-to-patient)**	165	10	155
**Other incident (not included in other categories)**	159	16	143
**Implementation of care and ongoing monitoring / review**	132	91	41
**Documentation (records, identification, and drug charts)**	131	82	49
**Consent, communication, confidentiality**	80	51	29

**Clinical assessment (diagnosis, scans, tests, assessments)**	55	40	15
**Medical device / equipment**	37	19	18
**Patient abuse (by staff / third party)**	27	2	25
**Infection Control Incident**	23	4	19
**Total**	**4112**	**1529**	**2583**
